# Exploring the reliability and acceptability of cognitive tests for Indigenous Australians: a pilot study

**DOI:** 10.1186/s40359-017-0195-y

**Published:** 2017-08-02

**Authors:** Kylie M. Dingwall, Allison O. Gray, Annette R. McCarthy, Jennifer F. Delima, Stephen C. Bowden

**Affiliations:** 1 Menzies School of Health Research, Institute of Advanced Studies, Charles Darwin University, PO Box 4066, Alice Springs, NT 0871 Australia; 2 Alice Springs Hospital, PO Box 2234, Alice Springs, NT 0871 Australia; 3 University of Melbourne, Melbourne, VIC 3010 Australia

**Keywords:** Indigenous, Aboriginal, Cognitive testing, Cognition, Cross-cultural

## Abstract

**Background:**

Reliable cognitive assessment for Indigenous Australians is difficult given that mainstream tests typically rely on Western concepts, content and values. A test’s psychometric properties should therefore be assessed prior to use in other cultures. The aim of this pilot study was to examine the reliability and acceptability of four cognitive tests for Australian Aboriginal people.

**Methods:**

Participants were 40 male and 44 female (*N* = 84) Aboriginal patients from Alice Springs Hospital. Four tests were assessed for reliability and acceptability – Rowland Universal Dementia Assessment Screen (RUDAS) (*n* = 19), PEBL Corsi Blocks (Corsi) (*n* = 19), Story Memory Recall Test (SMRT) (*n* = 17) and a CogState battery (*n* = 18). Participants performed one to three of the tests with repeated assessment to determine test-retest reliability. Qualitative interviews were conducted and analysed based on an adapted phenomenological approach to explore test acceptability. An Indigenous Reference Group gave advice and guidance.

**Results:**

Intra-class correlations (ICC) for test retest reliability ranged from *r* = 0.58 (CogState One Back accuracy) to 0.86 (RUDAS). Themes emerged relating to general impressions, impacts on understanding and performance, appropriateness, task preferences and suggested improvements.

**Conclusions:**

RUDAS, CogState Identification task, and SMRT showed the highest reliabilities. Overall the tests were viewed as a positive challenge and an opportunity to learn about the brain despite provoking some anxiety in the patients. Caveats for test acceptability included issues related to language, impacts of convalescence and cultural relevance.

## Background

Cognitive dysfunction may be prevalent among some Indigenous Australians due to the high rates of substance abuse, domestic violence, chronic illness, psychological stress or trauma, and malnutrition reported in this group [1]. Impairments in cognition including psychomotor, memory, attention, learning and executive functions have been reported for Indigenous Australians with chronic substance misuse [2, 3]. Limited access to healthy foods in remote regions leads to inadequate nutrition for some remote dwelling Indigenous people [4] and thiamine deficiency in particular results in the neurological condition Wernicke-Korsakoff’s Syndrome (WKS) [5, 6]. Wernicke’s encephalopathy, is an acute component of WKS characterised by mental confusion, ataxia, ophthalmoplegia and memory loss [5, 7, 8]. The chronic component, Korsakoff’s syndrome can occur if the acute deficiency is left untreated and manifests as chronic anterograde and retrograde amnesia, dementia-like impairment and less frequently disorientation, confabulation and lack of insight in severe cases [8–10].

Cognitive assessment enables the measurement of changes in brain function. In conditions such as WKS, repeated cognitive assessments may be required to monitor response to treatment and results can inform options for further clinical management. Test-retest reliability is important when monitoring cognitive progression to ensure that test results are consistent over time [11]. Learning effects or practice effects can occur where repeated exposure to the test improves subsequent performance due to practice or familiarity with test content [11]. It is therefore important to examine these psychometric properties when using cognitive tests to monitor change to ensure clinical decisions or research conclusions are based on reliable data.

Measuring cognition cross-culturally poses unique challenges when tests are based on Western cultural concepts [12, 13]. Existing cognitive tests can rely heavily on the use of the English language, require written responses and resemble mainstream educational processes [14]. Poor English literacy, a lack of formal education, as well as differing concepts of numbers, time and space can mean that Indigenous Australians may have limited experience with the knowledge base from which such tests are derived [1, 13, 15–19]. To address these issues, care should be taken to minimize cultural bias and the psychometric properties of the tests should be assessed within the population in which it is to be used.

Recent studies have proposed a number of priorities for selecting and designing appropriate tests for Indigenous Australians [20]. Such priorities include using tests with content, stimuli and formats that are relevant, familiar and engaging; with a decreased reliance on language, literacy and numeracy; have simple instructions; utilise prompts and feedback; are performance based where demonstrations and practice trials are used; and are portable and brief among other considerations [20]. Indigenous people themselves are best placed to determine relevance and acceptability of cognitive tests. Face validity refers to a participant’s perception of the test and whether, in their subjective opinion, it is a good test of what it purports to measure. If face validity and the acceptability of a test are low, a participant’s motivation to complete the test may be low, contributing to unreliable test scores [11].

The restricted aim of this pilot study was to examine the reliability and acceptability of four cognitive tests for monitoring change over time for Aboriginal people. Results from this study will inform test selection for a randomised controlled trial (RCT), monitoring cognitive outcomes following treatment for WKS.

## Methods

### Participants

Participants were a convenience sample of 40 male and 44 female (*N* = 84) Aboriginal patients from Alice Springs Hospital, recruited prospectively. Data collection occurred from March 2014 to December 2014. Participants originated from Alice Springs, Tennant Creek and remote communities across Central Australia, Western Australia and South Australia, representing 30 language groups. Inclusion criteria were expected admission for at least 48 h, 18 years or older, Aboriginal or Torres Strait Islander, able to communicate in English. Patients were excluded if they were pregnant. These criteria were used as the sample was intended to reflect the proposed sample for the subsequent RCT. Other exclusions for this study included identified pre-existing cognitive impairments, acute neurological conditions, under 18 years old, or unable to freely give informed consent. Seventy three follow up assessments were conducted. Some participants performed two (*n* = 12 participants) or three (*n* = 3 participants) cognitive tests during their admission due to their expressed interest to do so and to gain information on acceptability comparatively between tests. Five participants were lost to follow up for CogState (21.7%), three were discharged and two declined to continue. Four participants were lost to follow up for RUDAS (17.4%), two were discharged, one declined to continue and another was simultaneously recruited to a conflicting study.

Eight participants were lost to follow up for Corsi (29.6%), five were discharged and three declined further participation. Thirteen participants were lost to follow up for SMRT (43.3%), ten were discharged, two declined to continue and two had technical equipment failures.

### Materials/apparatus

The tests were selected based on assessment of cognitive domains affected in WKS and previous use in this population with use of culturally appropriate methodology. Testing was conducted in English as the tests were developed in this language, with the intention that the most suitable would be translated into key Aboriginal languages of the region for the RCT.

#### CogState (CogState)

CogState is a computerised test comprised of subtests that can be tailored to a specific research situation (www.cogstate.com) [2, 21, 22]. Minimal literacy is required to complete the test, and it has been used to assess cognition for Indigenous Australians in previous research [2, 23–27]. The battery used in this study consisted of 4 subtests (described below) and took approximately 20 min to administer [28]. The tests were fully supervised with brief on screen instructions also provided. Responses were recorded using the keyboard “D” and “K” keys for “no” and “yes” respectively. If a participant was left-handed the keys were reversed. The participant was allowed a short practice before each sub-task. This CogState battery assesses psychomotor speed function, visual attention, working memory and visual learning [28].

*The Detection Task (DET)* uses playing card stimuli presented onscreen to measure simple reaction time. The participant is required to press “yes” as soon as the card has turned face-up. This task measures visual attention and psychomotor function.

*The Identification task (IDN)* uses the same format as the Detection task to measure choice reaction time. Once the card turns over the participant is required to press “yes” if the suit is red or “no” if the suit is black. The identification task assesses visual attention.

*The One Card Learning task (OCL)* uses the same format and asks “Have you seen this card before?” The participant is required to attend to the cards as they appear and maintain each card in their working memory. When the card turns over, the participant decides whether it has been seen before in the current task. This task measures visual learning and memory.

*The One Back task (OBK)* asks “Is this card the same as the previous card?”. When the card turns over, the participant needs to determine whether it is the same as the last. This task measures attention (working memory).

Scores are provided in the form of log10 transformed mean reaction time (in milliseconds) for the detection and identification tasks and arcsine transformed accuracy (defined by number of correct responses divided by the total number of trials attempted) for the one card learning and the one back task.

#### PEBL Corsi block-tapping task (Corsi)

The original Corsi Block-Tapping test is a classic visuo-spatial working memory test used as a visuo-spatial version of digit span. A computerised version - Psychology Experiment Building Language (PEBL) of the Corsi was used in this study (see http://pebl.sourceforge.net/battery.html). Three practice attempts precede the scored testing. A flashing sequence of coloured squares is presented onscreen and the participant is required to replicate the pattern by touching the squares on the touch screen. The initial sequence begins with three squares and increases by one after each correct sequence. Participants are allowed only one incorrect attempt on each number of ‘blocks’. If two incorrect attempts are made for the same number of blocks, the test ceases. Total score is used for analyses.

#### Rowland Universal Dementia Assessment Scale (RUDAS)

The RUDAS is a short cognitive screening instrument designed to minimise the effects of cultural learning and language diversity. It was developed and validated for a culturally and linguistically diverse (CALD) population and has been translated into several languages [29–31]. RUDAS also assesses a broad range of cognitive functions, [29] and is valuable for assessing substance misuse related impairments [6, 32]. It generates an overall cognitive score based on measures of memory, body orientation, praxis, drawing, judgement, recall and language [30]. The RUDAS has been used extensively by the Addiction medicine team at Alice Springs Hospital and is considered the best available and a well-accepted cognitive mental status test for alcohol-related conditions in this clinical setting. RUDAS was administered and scored by a trained researcher according to the original administration guidelines. The first item, a memory recall task, requires learning and delayed recall of a four item grocery list. The body orientation task requires the participant to follow verbal instruction and point to specific body parts. The praxis item requires the participant to copy and continue an alternating hand movement. The visuo-constructional drawing item requires the participant to copy a picture of a cube. The judgement item asks what one does to get across the road safely. The final item requires the participant to state the names of as many different animals as they can within 1 min.

#### Story memory recall test (SMRT)

The SMRT is a modified version of the Wechsler Logical Memory Test. It requires participants to memorise a fictional passage that includes an accident or negative event and immediately recall the details [33, 34]. The test was chosen given the oral traditions and use of storytelling in many Aboriginal and Torres Strait Islander cultures [35]. An Aboriginal Project Officer developed the two locally relevant stories in English, in consultation with hospital Aboriginal Liaison Officers (ALOs) and the project’s Indigenous Reference Group. There were several revisions of the stories and their scoring guidelines to minimise repetition and to account for nuances in local vernacular.

With the participant’s consent, recall of the stories was audio recorded to ensure accurate scoring. Audio files were transcribed and scored as per the developed scoring guidelines where one point was allocated for each correct component recalled. The stories were scored by two raters and averaged with a total possible score of 24 for Story One and 21 for Story Two.

### Procedure

The Central Australian Human Research Ethics Committee (CAHREC) and the Human Research Ethics Committee of the Northern Territory Department of Health and the Menzies School of Health and Research (including the Aboriginal Ethics Sub-Committee) approved the study. Specifically trained researchers gained written informed consent prior to conducting the study.

After completing one of the cognitive tests, a short semi-structured interview was conducted with the participant to evaluate acceptability of the test and testing process. Participants were then asked to perform the same cognitive test 1–5 days later to replicate conditions for the RCT where retest would occur after 3 and 5 days. This also reflects changing needs in assessment practice, where decreased time and resources has increased demand for efficient clinical decision-making and average length of hospital stays have decreased [36]. While longer retest intervals may be desirable, we were primarily interested in alternative ranking of tests, evaluated in terms of short-term test retest reliability. Ranking of tests is not likely to change with longer retest intervals. Other researchers have demonstrated use of the CogState Battery at 10 min, 1 week and 1 month test-retest intervals where ICC results between assessments for OBK, DET and IDN tests (3–5) maintained reasonable reliabilities above 0.60 and raw difference values below 3% [37]. If participants were discharged or unwilling to complete the retest their quantitative results were excluded but interview data were retained in analyses.

Participants’ medical files were reviewed to record any relevant medical history, medication administration, length of hospital admission, pathology results, presenting diagnoses, and any recorded history of substance use or neurological impairment. Three participants did not consent to having their medical files reviewed.

### Statistical analysis

An alpha level of 0.05 was used for all statistical tests. Continuous variables are expressed as means and SDs and categorical variables are reported as percentages. Statistical analyses were conducted using IBM SPSS Statistics 22 [38].

ANOVA and Chi square statistics were used to assess for any demographic differences between the groups performing each test. To investigate retest reliability, ICCs for agreement and consistency were calculated.

Paired sample t-tests were used to examine any learning effects. To ensure that the pattern of statistical findings was not affected by distributional violations in small samples, a Wilcoxon signed rank test was conducted. The same pattern of results were achieved hence only the parametric analyses are reported.

The SMRT was scored by two raters (KD and AG) and results were used to calculate the ICC to examine inter-rater reliability. Inter-rater reliability examines how well scorers provide similar ratings and was calculated using a two way mixed, absolute, average measures ICC [39].

### Acceptability interview analysis

A simple transcendental phenomenological approach was utilised in developing interviews to explore the experience of Aboriginal Australian participants performing English-based cognitive tests. Initial discussions were held to bring awareness to the researchers’ preconceived assumptions, judgements, beliefs, perceptions and experiences [40] about the topic. These ideas were suspended during the process of bracketing [41, 42] before formulating interview questions for the study. Interviews were transcribed verbatim and analysed using NVivo 10 [43]. Four researchers initially evaluated the interview data independently for recurring themes (i.e. significant statements raised by more than one participant) for individual tests and to identify common themes across all the tests. The researchers discussed initial findings and agreed on a preliminary set of themes which were presented to the Indigenous Reference group for discussion. Data were then restructured and re-coded in order to answer the following underlying research questions in relation to participants’ experiences:

- What were the general impressions of the assessments?
- What impacted on understanding and performance?
- How appropriate were the tasks’ format and content?
- What would improve understanding and acceptability?
- Which tasks were preferred?

Some participant responses presented were edited for grammatical clarity. The first two authors participated in a secondary discussion about the revised structure and revised themes were agreed upon by consensus.

## Results

### Quantitative results

Demographic information for participants who performed both baseline and retest assessments are described in Table 1. There were no significant demographic differences between the groups performing each of the different tests.

**Table 1:** Comparison of retest study participants’ demographics by cognitive assessment

	CogState (*n* = 18)	Corsi (*n* = 19)	RUDAS (*n* = 19)	SMRT (*n* = 17)	F	df	*p* value	Effect size Ƞ^2^
Mean Age (SD)	40.65 (12.67)	46.33 (13.22)	48.45 (15.49)	49.81 (13.18)	1.55	3	0.21	0.06
Mean years Education (SD)	10.23 (1.83)	8.75 (2.38)	9.21 (2.08)	9.27 (2.20)	1.09	3	0.36	0.07
Mean no. of languages spoken (SD)	2.39 (1.20)	2.42 (1.43)	2.26 (0.99)	2.65 (1.06)	0.32	3	0.81	0.01
					χ^2^	df	*p* value	Effect size Phi
N with English as first language (%)	7 (38.88%)	7 (36.84%)	6 (31.57%)	8 (47.05%)	0.93	3	0.817	0.11
N Males (%)	6 (33.33%)	6 (31.57%)	8 (42.10%)	10 (58.82%)	3.37	3	0.337	0.22

Primary and secondary diagnoses were recorded from the file audit using ICD-10 coding and are presented in Table 2.

**Table 2:** Study participants’ primary and secondary diagnoses defined by International Classification of Disease – 10 coding system

ICD-10 chapter	ICD-10 categories	Primary code	Secondary code
I Certain infectious and parasitic diseases	A00-A09 Intestinal infectious diseases	1	1
	B35-B49 Mycoses	1	
	A50-A64 Infections with a predominantly sexual mode of transmission		1
	B15-B19 Viral hepatitis		2
	B65-B83 Helminthiases		1
	B85-B89 Pediculosis, acariasis and other infestations		1
	B95-B98 Bacterial, viral and other infectious agents		10
II Neoplasms	C00-C97 Malignant neoplasms	1	1
	D10-D36 Benign neoplasms	1	
III Diseases of the blood and blood-forming organs and certain disorders involving the immune mechanism	D60-D64 Aplastic and other anaemias		3
	D65-D69 Coagulation defects, purpura and other haemorrhagic conditions		3
IV Endocrine, nutritional and metabolic diseases	E00-E07 Disorders of thyroid gland		1
	E10-E14 Diabetes mellitus	1	49
	E20-E35 Disorders of other endocrine glands		1
	E65-E68 Obesity and other hyperalimentation		2
	E70-E90 Metabolic disorders	3	13
V Mental and behavioural disorders	F10-F19 Mental and behavioural disorders due to psychoactive substance use		3
VI Diseases of the nervous system	G40-G47 Episodic and paroxysmal disorders		1
VII Diseases of the eye and adnexa	H00-H06 Disorders of eyelid, lacrimal system and orbit		1
IX Diseases of the circulatory system	I00-I02 Acute rheumatic fever	1	
	I05-I09 Chronic rheumatic heart diseases		2
	I10-I15 Hypertensive diseases		15
	I20-I25 Ischaemic heart diseases		1
	I26-I28 Pulmonary heart disease and diseases of pulmonary circulation	1	1
	I30-I52 Other forms of heart disease	5	2
	I80-I89 Diseases of veins, lymphatic vessels and lymph nodes, not elsewhere classified		2
	I95-I99 Other and unspecified disorders of the circulatory system		3
X Diseases of the respiratory system	J09-J18 Influenza and pneumonia	1	
	J20-J22 Other acute lower respiratory infections		1
	J95-J99 Other diseases of the respiratory system		1
	J40-J47 Chronic lower respiratory diseases	1	
XI Diseases of the digestive system	K00-K14 Diseases of oral cavity, salivary glands and jaws		1
	K55-K64 Other diseases of intestines	1	1
	K70-K77 Diseases of liver		1
	K80-K87 Disorders of gallbladder, biliary tract and pancreas	1	
	K90-K93 Other diseases of the digestive system	1	1
XII Diseases of the skin and subcutaneous tissue	L00-L08 Infections of the skin and subcutaneous tissue	5	3
	L60-L75 Disorders of skin appendages	1	
	L80-L99 Other disorders of the skin and subcutaneous tissue	1	
XIII Diseases of the musculoskeletal system and connective tissue	M00-M25 Arthropathies	2	3
	M40-M54 Dorsopathies		3
	M60-M79 Soft tissue disorders	1	3
	M80-M94 Osteopathies and chondropathies	1	2
XIV Diseases of the genitourinary system	N00-N08 Glomerular diseases		2
	N10-N16 Renal tubulo-interstitial diseases	1	
	N17-N19 Renal failure	1	21
	N80-N98 Noninflammatory disorders of female genital tract		3
XVIII Symptoms, signs and abnormal clinical and laboratory findings, not elsewhere classified	R00-R09 Symptoms and signs involving the circulatory and respiratory systems		2
	R10-R19 Symptoms and signs involving the digestive system and abdomen	1	5
	R25-R29 Symptoms and signs involving the nervous and musculoskeletal systems		1
	R30-R39 Symptoms and signs involving the urinary system		1
	R50-R69 General symptoms and signs	2	7
	R70-R79 Abnormal findings on examination of blood, without diagnosis		1
	R90-R94 Abnormal findings on diagnostic imaging and in function studies, without diagnosis		2
XIX Injury, poisoning and certain other consequences of external causes	S70-S79 Injuries to the hip and thigh	2	
	S80-S89 Injuries to the knee and lower leg	1	
	T20-T32 Burns and corrosions	1	1
	T80-T88 Complications of surgical and medical care, not elsewhere classified	3	2
XX External causes of morbidity and mortality	V01-X59 Accidents		5
	Y40-Y84 Complications of medical and surgical care		5
XXI Factors influencing health status and contact with health services	Z30-Z39 Persons encountering health services in circumstances related to reproduction		1
	Z40-Z54 Persons encountering health services for specific procedures and health care	4	
	Z55-Z65 Persons with potential health hazards related to socioeconomic and psychosocial circumstances	1	1
	Z70-Z76 Persons encountering health services in other circumstances		16
	Z80-Z99 Persons with potential health hazards related to family and personal history and certain conditions influencing health status		19
ZZ	Not Listed	5	7
	Total	53	242

**Fig. 1:**
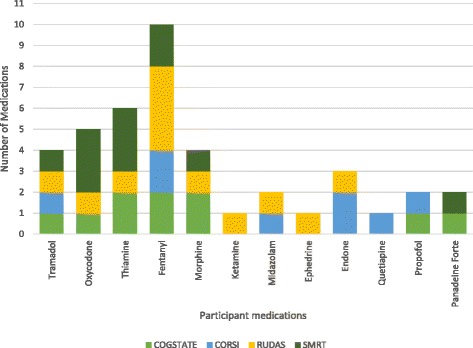
File audit of number of participant medications across each cognitive test. CogState participants had 10 notations of medication administration, Corsi participants had eight, RUDAS had 12 and SMRT had 11 medication administrations

All participant medications were recorded and summarised into classes and are described in Fig. 1.

Table 3 presents the means and standard deviations for each task at baseline and retest and results of the paired samples t-test. There were no significant differences between baseline and retest scores except for Story One on the SMRT which demonstrated significant improvement from baseline to retest with a moderate effect size.

**Table 3:** Baseline and retest means, standard deviations and paired sample t-test results

Test	Baseline		Retest		Paired t-test	Effect Size
	Mean	SD	Mean	SD		Ƞ^2^
CogState						
DET RT	2.69	0.21	2.63	0.17	t(17) = 1.52, *p* = 0.15	0.08
IDN RT	2.80	0.12	2.78	0.14	t(17) = 0.71, *p* = 0.49	0.04
OCL ACC	0.90	0.12	0.94	0.17	t(17) = −1.17, *p* = 0.26	0.06
OBK ACC	1.10	0.30	1.20	0.26	t(17) = −1.69, *p* = 0.11	0.09
Corsi	37.44	14.57	41.33	14.98	t(17) = −1.34, *p* = 0.20	0.07
RUDAS	24.95	3.97	25.68	4.21	t(18) = −1.49, *p* = 0.15	0.08
SMRT						
Story 1	8.62	4.48	11.00	4.54	t(16) = −2.98, *p* = 0.01	0.16
Story 2	9.44	3.52	9.82	4.30	t(16) = −0.51, *p* = 0.62	0.03
Total	18.06	7.04	20.82	8.01	t(16) = −2.15, *p* = 0.05	0.23

*Note:* RT = reaction time, ACC = accuracy, Total = Story 1 + Story 2

ICCs for the four cognitive assessments are presented in Table 4. ICC results ranged from CogState OBK ACC (0.58) to RUDAS total score (0.86). ICCs are presented for inter-rater reliability in Table 5 for the SMRT and indicate excellent agreement between the two raters with all ICCs above 0.98. Average time taken for each test is presented in Table 6.

**Table 4:** Test retest reliability based on two-way random, intra-class correlation coefficients for consistency and agreement between baseline and retest

Test	ICC consistency	95% CI	ICC agreement	95% CI
CogState DET RT (*n* = 18)	.64^*^	.26 – .85	.63^*^	.23 – .84
CogState IDN RT (*n* = 18)	.83^**^	.61 – .93	.84^**^	.62 – .94
CogState OCL ACC (*n* = 18)	.63^*^	.41 – .91	.63^*^	.26 – .84
CogState OBK ACC (*n* = 18)	.60^*^	.31 – .90	.58^*^	.19 – .82
Corsi Total Score (*n* = 18)	.65^**^	.28 – .85	.64^**^	.28 – .85
RUDAS Total Score (*n* = 19)	.86^**^	.68 – .94	.85^**^	.66 – .94
SMRT Story 1 (*n* = 17)	.72^**^	.38 – .90	.70^**^	.30 – .87
SMRT Story 2 (*n* = 17)	.77^**^	.47 – .91	.74^**^	.40 –.90
SMRT Total Story 1+ Story 2 (*n* = 17)	.75^**^	.44 – .90	.72^**^	.36 – .89

^*^ *p* < .05; ^**^ *p* < .01

**Table 5:** Inter-rater reliability analysis based on Intra-class correlations between scorers for SMRT at baseline and retest

Test	Baseline ICC	Retest ICC
SMRT Story 1	0.98	0.99
SMRT Story 2	0.99	0.99
SMRT Total (Story 1+ Story 2)	0.99	0.99

**Table 6:** Mean (SD) total time to complete each of the cognitive tests

Test	Baseline mean time in minutes (SD)	Retest mean time in minutes (SD)
CogState	13.28 (2.97)	10.55 (2.48)
Corsi	4.16 (1.78)	3.49 (0.82)
RUDAS	7.45 (2.58)	6.55 (1.75)
SMRT	4.5 (1.83)	4.20 (1.80)

### Observations: interruptions and distractions

Some Interruptions and distractions were also observed to occur during testing and are inevitable in this acute clinical setting. The hospital environment was often loud with high potential for distractions including: other patients, nursing checks, visitors, machine alarms, television volume, phone calls and medication administration. Test administrators used their professional judgment at the time of testing to determine whether the test session had been compromised and if a test needed to be re-taken. More of these issues were recorded for the computerised assessments. The non-computerised tests had nine instances of interruptions and distractions (i.e. noise) whereas the computerised tests had 19 observations noting these. RUDAS had five recorded interruptions or distractions, the SMRT had four. Corsi had seven and CogState had 12 interruptions/distractions noted.

### Acceptability interview results

#### General impressions

##### A good challenge

Most participants were generally open to performing the assessments and some identified them as ‘fun’ and a good way of exercising the mind. All assessments were described by participants as challenging, but this was often seen in a positive light.

“Liked that it gave me a bit of a muddle in my brains.” P9 Corsi

Some tasks, particularly the computerised tasks, were perceived to be easy at first, but then increased in difficulty causing confusion, or feelings of frustration. Some did not like the length or speed of the tests as this was perceived to increase difficulty. Nevertheless the tasks appeared to provoke the desired response of motivating people to do well.

“Not bad, like playing a game but it got too hard.” P17 Corsi

“Easy test. Started out easy, and then got hard. Was a good challenge.” P12 Corsi

“I did not like that I got some wrong as I enjoy challenging mind games.” P20 CogState

##### Performance Anxiety

Many people felt concerned that they may not perform well on the test and were worried what might happen if they did get a wrong answer or press the wrong button. They also suggested others might feel shy when asked to perform the test and some commented on feelings of being ‘judged’ or as if their ‘intelligence was being questioned’. Some participants therefore required constant reassurance to continue performing the test despite good performance.

“It is important that we explain to people that they don’t have to get the right answer all the time. Aboriginal people may think if I push the wrong button am I going to get in trouble? Explaining that it’s just a game will put people at ease.” P22 CogState

“Some people would be shy if asked to do the test on their own and would perform better if we get a few of them together.” P25 RUDAS

Others suggested it was easier to recall the story with friends and family later, than with the researcher. One participant acknowledged that “it is a test and we all get frustrated and nervous… Being nervous is a natural part of life and this anxiety is channelled into test performance*” P4 CogState.* Others, despite feeling anxious also felt “determined*” P38 SMRT*.

##### Memory and an opportunity to learn or improve

Memory was a topic frequently mentioned during the interviews. It was perceived that assessments would help improve memory and performing the test provoked memories for several participants, some of whom related the tasks to daily activities.

[The visuospatial orientation task is] “like a small child learning their left and right.” P28 RUDAS

“I might buy these things [in the shopping list task]. I cook all those at home.” P57 RUDAS

“Test made me frustrated. It made me think back to when I used to drink …The test improves my mind and brings back memories from years ago. This could help heal people’s minds and help bring memories back.” P29 CogState

Participants therefore viewed the assessments as an opportunity to learn about their minds, a means to keep busy, to learn about computers and increase awareness of brain function. Some thought it was good to test their brains due to situations such as substance use, being a victim of violence and forgetfulness.

“An activity like this is good; it keeps you tuned into what your brain does.” P4 SMRT

“This activity will teach people about their minds which will help people understand the damage alcohol does to the brain.” P29 CogState

The tests therefore made participants reflect upon their own situations and performance, particularly for the SMRT, seemingly because it made poor performance explicit to participant themselves. As a result some participants felt a need to justify or explain their performance.

“Hard to remember the exact words. I got mixed up and blank.” P40 SMRT

“I felt like a failure. I couldn’t say the same sentence precisely, but I knew what the story was about.” P45 SMRT

#### Impacts on understanding and performance

When asked how other Aboriginal people would perform on these assessments, most respondents felt that they would perform reasonably well. A few participants were concerned that older Aboriginal people or those with English as a second language would struggle with the assessments due to lack of familiarity with the testing process and certain language concepts.

“Older people would struggle using the computer and understanding the test in English.” P26 CogState

##### Language

Unsurprisingly language was overwhelmingly cited by participants as a factor that may influence understanding of the test. Despite using plain English, some items were met with silence and some participants required prompting, repetition or clarification of instructions. A number of participants were also concerned about word usage and pronunciation affecting their understanding and performance.

“If people know English they will do well in the test.” P32 CogState

“I only know plain English. A bit hard to pronounce.” P67 SMRT

“Get someone from community to talk and liaison explain it. Cause he won't understand yous two.” P9 Corsi

Some suggested that there may not be words for certain things in language, which might contribute to confusion and lack of clarity in instructions.

“Not sure if there is a word for left hand side, right hand side in language. I never tried it.” P25 RUDAS

Others, some of whom admitted that they spoke English well, found the instructions clear and easy to follow, and others found the use of picture prompts to be useful.

“My husband was a white fella and I understand English.” P25 RUDAS

“Easy to understand when you say blocks go yellow then point it out with the piece of paper” [screenshot]. P9 Corsi

##### Education

Similarly, some participants believed that a lack of education made the activity challenging and those with more education would find the activity easier.

“Just those numbers, make sure he knows his numbers... If he don’t know his numbers he’ll miscount.” P9 Corsi

“Lack of schooling made this activity hard.” P11 Corsi

“Young ones with mainstream learning would be ok.” P16 Corsi

##### Illness

Participants expressed concern about their convalescence and its potential impacts on their performance. Their capacity to think and concentrate was monopolised by their illness or social situations. Participants discussed substance abuse, stroke episodes and revealed head injuries due to domestic violence as concerns for their memory.

“…I am thinking about other things… I followed your words, but I picked up others. I like it but not really too good at it because I am sick at the moment.” P8 SMRT

“Testing the memory, refreshing yourself, but the hospital is not a good place to do it because patients are sick.” P39 SMRT

One participant was concerned that his “mind goes blank” due to his use of marijuana. “I’m not in my right mind. If I was in my right mind I would be able to remember the whole story.” P46 SMRT. There were physical impediments such as limbs in casts or traction, medical equipment and intravenous fluid lines that may have influenced participant performance on physical aspects. For example, IV cannulation and being connected to dialysis impacted on the ability to form an upright fist on the RUDAS praxis task for a couple of participants and IV lines and plaster casts impacted on using ‘yes’ and ‘no’ keys in Cogstate (which required both hands) for other participants. A few participants also expressed concern for others with vision or hearing problems.

“Other Aboriginals would find this test hard, especially those with hearing impairment.” P8 RUDAS

[Make] “the pictures bigger. Some people probably can’t see properly much.” P76 CogState

#### Appropriateness of the task format and content

##### Computerised format

Some participants seemed comfortable using the computerised tests despite varying levels of education and exposure to technology and likened the experience to games on their mobile phones.

*“*Not a lot of Aboriginals have access to computers. Make a smart phone app instead of a computer because people are more familiar with phones.*”* P22 CogState.

Some participants who performed multiple tests stated they preferred the computer-based assessments as opposed to the verbal tests. Participant P22 (CogState) also mentioned that “some Aboriginal people may not want to do testing person-to-person because they don’t trust their information will be used appropriately…with the computer you know it’s not going to be flung back in your face.”

##### Familiarity

It was noted that perceptions of task appropriateness may change in accordance with content familiarity, location and diversity of different population groups. SMRT stories appeared most relevant to people who reside in or have knowledge of remote areas. CogState appeared most relevant to people who actively played card games. Overall, the computer-based assessments seemed more relevant to those who had used computers.

“…Not sure if Aboriginal people from other places will relate to the stories.” P39 SMRT

“I didn't learn cards… The ones that play cards would like this game.” P79 CogState

“Some people would find this confusing and hard to understand the instructions due to language barrier, low level of education and also elderly people who are not used to computers.” P17 Corsi

##### Cultural relevance

Cultural relevance and relatability influenced perceived appropriateness of the task. The SMRT stories, describing aspects of remote community life, were generally considered relevant. Their content invoked memories and comparisons to everyday life. The process of reciting stories is common practice in Aboriginal culture however a number of participants appeared not to enjoy being tested on this skill.

“Story game was interesting as the story was realistic…Good stories and grateful to read them…The story is a good way of checking memory because it is real life.” P42 SMRT

“What the stories say, our people, that's what we do. We go out hunting and all those things, you know. It's good.” P67 SMRT

“Good. We listen to a whole lot of stories all the time and we can remember them pretty good.” P38 SMRT

Conversely, three participants questioned the authenticity of the content and format.

“The team would fix the tyre ‘blackfella style’ by tying their shirts around the wheel because they would not have missed the game.” P41 SMRT

“Stories ok. Made me laugh a bit. Was chased by the dingoes. Must be different community. Because we always go hunting and the dingoes run away from us but he was chased by the dingoes.” P51 SMRT

Participants were sometimes frustrated with the repetitive nature of some of the assessments. There were particular concerns with repeating the shopping list task of the RUDAS up to five times. Another participant was concerned about repeating words of the SMRT verbatim as it wasn’t a process routinely performed in Aboriginal culture.

“I don't like talking the same thing over and over. Some people would get angry if asked to repeat over and over. People would remember the shopping list, even if they didn't repeat it a few times.” P25 RUDAS

“Most Aboriginals paraphrase and leave out details that aren't important, even if they actually know those minor details…Older Aboriginal people can't remember names and modern things but they are unbelievable at remembering landmarks and traditional things.” P33 SMRT

“The tasks were not easier and still repetitive this time round…” P4 CogState

In discussion about specific tasks it was apparent that context and worldview influences how a question is interpreted and answered. Participants made comparisons to personal experience and suggestions to improve the delivery.

“Most people do not buy eggs. This is hard to remember. Everyone buys flour, tea, sugar, milk and bread.” P9 RUDAS

A number of participants asked for the stories to be re-read as cultural practices are often guided by repetition in storytelling.

“…In corroboree people follow from one point to a second point but they may repeat that in a song up to three times. That is why a lot of older people will memorise it that way.” P43 SMRT

A few participants mentioned that they would prefer to do the testing in a different environment.

“Would be better outside or out bush.” P81 RUDAS

##### Length and Speed

The length of some of the tests was a problem for a number of participants who thought that some of the stories were too long and that CogState felt like it was “dragging on” P32. This contributed to some boredom and loss of concentration, particularly for the CogState test. One person however suggested that the stories should be made “a bit longer, so it can sink in” P82 SMRT. A few participants also commented on the speed of some of the tests being too fast to keep up.

“It was quick and you need to be alert so that you can remember. I got to the point where I was guessing because the cards were coming too quick and too fast to retain. Feelings of frustration.” P4 CogState

#### Suggested Improvements

##### Introduction process

Aspects of the introduction process were mentioned as potential influences on understanding the test. Participants described the importance of introducing and explaining the assessments appropriately. It was observed that participants wanted to be prepared or relaxed, and gain a true understanding of the task to be performed. Suggestions for improvement in explaining the tasks to others therefore included providing enough warning to mentally prepare, use of pictures and physical cues (e.g. hand signals, actual cards) and ensuring a comprehensive explanation delivered in plain English or language. Providing opportunities for practice or ‘warm ups’ and repetition of the story scripts were other suggestions raised.

“A really big story like that, you need to relax first. Because if you’ve got a lot of things in your mind, going round and round you know, you can’t really think about what happened…” P88 SMRT

“If I had to explain it, show picture first, test-run, and explain it step by step.” P12 Corsi

“You could use actual cards. Aboriginal people have seen cards throughout their lives. They know what they are about.” P22 CogState

##### Interpreters

Language was mentioned as an obvious factor to consider ensuring appropriate communication and understanding. The general consensus was that instructions in their first language, use of an interpreter and ALOs would assist understanding and improve performance.

“Some sort of interpreter and all that. So that they would know. So they can understand properly. Some are misunderstanding.” P95 CogState

“Use ALOs to help. The introduction to this activity is important and that people should understand why they are doing the test.” P17 Corsi

Conversely, a few participants noted that the adapted plain English instructions were sufficient.

“Not hard, easy sentences.” P37 SMRT

“I think they could follow it quite easily without too many problems [the practice] is simple and clear.” P13 Corsi

##### Format and content changes

There were a number of proposals for altering the format or content of tasks. Participants made suggestions such as use of physical objects (playing cards) to make the task more concrete or relevant.

“Actually sit down and play the game with them using cards.” P32 CogState

Other suggestions included to incorporate content such as football and other sports, group testing so the person didn’t feel singled out and have other options to record answers (apart from audio on SMRT). Several participants confirmed that the pictorial resources provided (flipchart and screenshot of testing platforms) were helpful in gaining understanding of the task.

“The flip chart is good. Pictures are better (than words)… People feel scared to consent to audio because they don’t know what they are going to say. Give them the option to write or record their answers.” P12 Corsi

“You could put pictures behind the blocks (shapes and animals) so that people can remember what’s behind it.” P19 Corsi

#### Task preferences

Participants were questioned about individual subtasks of the SMRT, CogState and RUDAS to see if there were any favoured tasks. Those that performed multiple tests were also asked if they preferred one of the assessments over another. A few participants stated that they did not have preference for any of the assessments performed. Generally, if a participant performed both a verbal and a computerised test, they preferred the computer-based testing. The SMRT appeared to be the least popular amongst participants, seemingly because it was more difficult and made any impairment explicit to patients themselves.

“Hard to remember the exact words. I got mixed up and blank.” P40 SMRT

“I felt like a failure. I couldn’t say the same sentence precisely, but I knew what the story was about.” P45 SMRT

Once the procedure was described there was some reluctance at the idea of the SMRT or refusal to proceed. Participants were given the option of another cognitive test or to withdraw from the study. One third of participants in the study declined to have their voice recorded on the consent form suggesting this format was not acceptable to some. Participant P12 (Corsi) suggested that “people feel scared to consent to audio because they don’t know what they are going to say. ” Another, Participant P79 (SMRT) was willing to complete the SMRT but not have their voice recorded. Their recollection of the story was instead transcribed onto paper. One participant seemed to feel the need to defend their performance on the story recall, but seemed happier with the CogState performance.

It was observed that some participants appeared to lose concentration, become tired and exhibited signs of boredom whilst conducting the CogState test. Whereas others preferred CogState or Corsi over the other tasks performed.

“The card one easier for most people.” P15 CogState and Corsi

“Prefer Corsi Block test over CogState because it is easier to remember.” P20 CogState and Corsi

“CogState over the story because I like to learn about computer. The story game was harder than the CogState.” P28 CogState and SMRT

“I liked the game on the computer better.” P72 CogState and RUDAS

Whilst some parts of the RUDAS were enjoyed by participants, other parts were confusing, disliked or perceived as patronising. For example, participants generally seemed to enjoy the animal task of RUDAS but not repeating the shopping list. The judgement task was also seen as confusing for some.

“I liked the animal part.” P1 RUDAS

“The road question would be a bit funny for other Aboriginals.” P24 RUDAS

[I didn't like] “repeating the shopping list. I don’t like talking the same thing over and over. Some people would get angry if asked to repeat over and over.” P25 RUDAS

## Discussion

This study is one of very few to explore the psychometric properties and acceptability of cognitive tests in an Aboriginal adult population. Results demonstrated reasonable retest reliabilities ranging from 0.58 to 0.86, good inter-rater reliability at 0.98 to 0.99 for the SMRT and minimal learning effects. Test-retest reliability of 0.70 is thought to be adequate for early stages of research, while reliability for experimental group research should be above 0.80 and the standard for individual assessment and clinical decision making should be at least 0.90 [44]. Interview data suggested that the tests demonstrated face validity as they were seen as an opportunity to “teach people about their minds” and “a good challenge.” However, as in other populations, the tests also provoked anxiety and uncertainty about the purpose, despite the use of detailed explanations and introductions. Participants discussed content, format and process familiarity as influences on task appropriateness. As anticipated, language, social situations and convalescence were potential impacts on participants’ understanding and performance. Use of Aboriginal languages, pictures or hand signals during instruction was proposed to improve understanding.

Generally the computerised tests (Corsi and CogState) demonstrated lower retest reliabilities than the non-computer based tests (RUDAS and SMRT), although they were not statistically significantly different. Low reliabilities for computerised tests is somewhat unusual given the purported increased reliability of computerised tests due to their standardised administration, ability to record many responses in a short period, multiple alternate forms and automatic standardised scoring [21, 45]. The modest reliabilities for Corsi and CogState subtasks (DET, OCL and OBK) may be attributable to the lack of familiarity with computers within this group as other studies have demonstrated an effect of computer familiarity on task performance [24]. This interpretation was supported by the interview data. In addition, the computerised tests had increased potential for interruptions as they were slightly longer than the non-computerised tests and maintained a predetermined, fixed administration pace which could not be paused. The average administration for the four CogState subtasks was 13 min - the longest test, and subsequently exhibited the most number of interruptions. To avoid these issues in the future, signage could be used requesting staff to avoid interruptions, a dedicated assessment room might be sought, testing could occur at nominated ‘quiet’ times or the number of subtasks might be reduced.

The Identification subtask of CogState demonstrated higher reliability than the other computerised tasks and one of the highest retest reliabilities overall (*r* = 0.84). This task is relatively easy to comprehend, compared to other CogState tasks, and it was second in the battery so it is possible that participants had time to become familiar with the testing platform, develop a strategy and also experience a decrease in test anxiety [46]. The third task, One Card Learning, was mentioned by participants as difficult and lengthy. Increased complexity may have contributed to reduced reliability in the latter tasks. The length of time taken to complete the test might also have impacted participants’ motivation. Boredom and a decline in concentration were suspected in some participants particularly for the one card learning task as it appeared to take the greatest length of time. Of course, as interpreters were not available and English was generally not a first language for participants, there is also a chance that participants did not fully comprehend these latter, slightly more complex tasks despite their apparent capacity to communicate in English.

The SMRT was selected for its relevance to the oral traditions and use of storytelling in Indigenous cultures. However, the strict and unfamiliar test requirements and associated performance anxiety, along with inability to access interpreters may have produced the more modest reliability compared to similar logical memory tests in other studies (e.g. *r* = 0.98) [47]. A practice effect was observed for Story One but was not observed for Story Two and was no longer significant when the total of Story One and Story Two was used. This effect may suggest that any unfamiliarity with task requirements, that might impact performance, diminishes by the second story. The inter-rater reliability coefficients indicated that any variance due to different raters is trivial. High inter-rater reliability suggests the comprehensive descriptions within the scoring guidelines led to objective and consistent rating of elements across raters. While the reliabilities achieved in this study were acceptable for use of the test in a research setting, the test’s validity to rule in or rule out disease states has not yet been demonstrated in this population. Further research is needed to confirm this.

The RUDAS demonstrated the best reliability of all four tests (*r* = 0.86) however, it was not as high as that originally obtained by the test developers (*r* = 0.98). This result may have occurred due to demographic characteristics and geographical differences in the sample of participants, (i.e. rural Aboriginal participants compared to urban participants of varied cultural background). Additionally despite a lack of alternate forms, no practice effects were observed for this test suggesting increased exposure to the same test stimuli did not impact on test performance, making this an appropriate test for repeated assessment in this setting.

Factors related to test delivery, format, and content identified in previous research were also identified as factors that impacted on acceptability of the cognitive tests in this study. As expected, language was an important factor impacting on understanding and use of interpreters was a proposed solution. However, use of an interpreter can introduce subtle variations to test administration and scoring. According to RUDAS developers [31] a briefing should occur with interpreters before conducting the assessment, highlighting the importance of precise interpretation of instructions and participant responses and requesting the interpreter note occasions where participant performance may have been affected by changes to the test due to language or cultural factors.

Attempts to engage translators in preparation for this study and later attempts to translate the tests into Aboriginal languages highlighted the difficulties with this process in maintaining test integrity. Even when trained interpreters are used, ensuring equivalent meaning where particular words may not exist is difficult and has the potential to equally impact on test reliability. Study inclusion criteria included ability to communicate in English, however this was based on nursing staff opinion rather than more objective measures. The lack of assessment of language ability and understanding, subjective measures to determine interruption impact during testing and absence of interpreter use are considered limitations in this study. A small sample size has contributed to wide confidence intervals obtained and can also be considered a limitation. Nevertheless, this situation potentially reflects ‘real world’ conditions as qualified interpreters are not always utilised, especially where English comprehension is perceived as adequate. To increase cultural safety, ALOs or the Aboriginal Interpreter Service could be consulted for participant introductions and to aid in building rapport. Regardless, the issue of availability of trained, adequately qualified interpreters remains a challenge in this context but it is somewhat encouraging that reasonable reliabilities can be observed without the use of interpreters in this study. Future research could examine whether reliability increases with appropriate language modifications or translations.

A preference for concrete explanations was apparent in participant suggestions for improving instructions. Harris and Harris [48] discuss views on Aboriginal learning styles and suggest a preference for knowledge based on a tangible context as opposed to abstract principles and to observe rather than be guided by oral or written instruction. This is reflected in participants’ suggestion to use pictures, then practice and then explanation of the instructions and that physical cues be used where appropriate (e.g. show the computer or actual cards). They also suggested an increased use of pictures or hand signals. These suggestions have previously been used as a means to reduce effects of language and cultural differences [49].

While test anxiety is potentially common across cultures, anxieties identified in our group may have been amplified by a historical misuse of assessment as a process of social and cultural control, leading to suspicion and mistrust of the process among many Indigenous Australians [50]. Interestingly, some participants felt less threatened when completing assessments on the computer, suggesting it was less likely to be “flung back in your face”. They also stressed the importance of putting people at ease, and suggested letting people know that they will not get in trouble and that it is ok if they make a mistake. Establishing rapport with participants and fully explaining the test and its purpose prior to testing are advised by the Australian Psychological Society to improve acceptability and cultural safety [51]. Nevertheless, cognitive testing in Australia is still typically based on Western concepts and values. It is therefore important to have a sound understanding of the test, including its limits when used with Indigenous people [51].

## Conclusion

This study indicated that the RUDAS, CogState identification task and the SMRT demonstrated the most adequate retest reliabilities for research purposes [44]. These tests will be employed in the upcoming RCT. Overall the tests were viewed as a positive challenge, an opportunity to learn about the brain and reflect on one’s own situation. Considerations for test acceptability included the use of interpreters, impacts of convalescence and cultural relevance. Results reflected previous studies recommending use of tests with: (1) content, stimuli and formats that are relevant, familiar and engaging, (2) a decreased reliance on language, literacy and numeracy, (3) simple instructions, (4) prompts and feedback, (5) are performance based where demonstrations and practice trials are used, and (6) are portable and brief [20].

## Abbreviations

ALOs: Aboriginal Liaison Officers
ANOVA: Analysis of variance
CAHREC: Central Australian Human Research Ethics Committee
CALD: Culturally and linguistically diverse
CogState: CogState battery
Corsi: PEBL Corsi Block-Tapping Task
DET: Detection task
ICC: Intraclass correlation coefficient
IDN: Identification task
OBK: One back task
OCL: One card learning ﻿task
RUDAS: Rowland Universal Dementia Assessment Scale
SDs: Standard deviations
SMRT: Story memory recall test
